# Real-Time Monitoring of HT-PEMFC

**DOI:** 10.3390/membranes12010094

**Published:** 2022-01-15

**Authors:** Chi-Yuan Lee, Fang-Bor Weng, Chin-Yuan Yang, Chun-Wei Chiu, Shubham-Manoj Nawale

**Affiliations:** Yuan Ze Fuel Cell Center, Department of Mechanical Engineering, Yuan Ze University, Taoyuan 32003, Taiwan; fangbor@saturn.yzu.edu.tw (F.-B.W.); doris.yang@mdpi.com (C.-Y.Y.); aa487156aa@gmail.com (C.-W.C.); shubham.nawale86@gmail.com (S.-M.N.)

**Keywords:** HT-PEMFC, flexible three-in-one microsensor, real-time microscopic monitoring

## Abstract

During the electrochemical reaction of a high temperature proton exchange membrane fuel cell (HT-PEMFC), (in this paper HT-PEMFC means operating in the range of 120 to 200 °C) the inhomogeneity of temperature, flow rate, and pressure in the interior is likely to cause the reduction of ion conductivity or thermal stability weight loss of proton exchange membrane materials, and it is additionally likely to cause uneven fuel distribution, thereby affecting the working performance and service life of the HT-PEMFC. This study used micro-electro-mechanical systems (MEMS) technology to develop a flexible three-in-one microsensor which is resistant to high temperature electrochemical environments; we selected appropriate materials and process parameters to protect the microsensor from failure or damage under long-term tests. The proposed method can monitor the local temperature, flow rate, and pressure distribution in HT-PEMFC in real time.

## 1. Introduction

A fuel cell is an important electrochemical device, which can directly convert the chemical energy of fuels such as methanol and hydrogen into electrical energy [1,2,3]. Unlike lithium-ion batteries and flow batteries, fuel cells have the advantages of high efficiency and low pollution [4,5]. The main electrochemical active materials in PEMFCs are all gas phases, and the only product produced is water, which is an absolute green energy technology. PEMFCs using polymer electrolytes to separate anode and cathode have gained especially numerous scientific and engineering interests.

With the depletion and pollution of fossil energy, the demand for efficient and clean energy is increasing. PEMFCs have attracted more and more attention because of their good conversion efficiency, environmental characteristics, simple structure, and low noise [6]. Under different working temperatures, PEMFCs can usually be divided into low-temperature PEMFCs and high-temperature PEMFCs. The energy density of HT-PEMFC is slightly lower than that of low temperature PEMFCs. Low temperature PEMFCs are easily polluted by carbon monoxide in syngas, and they require complex heat and water management systems. HT-PEMFCs can work at temperatures above 100 °C without complicated water and heat management systems, and they have good carbon monoxide tolerance [7,8]. Therefore, in recent years, research has focused more and more on HT-PEMFCs.

With regard to fuel cell products, they have been developed and widely investigated for their applications within mobile phones, notebook computers, stackers, electric bicycles, automobiles, and buses, among other uses. Although the developmental potential of fuel cells has attracted much attention, their high-cost issues, such as water management and the expensive Pt catalyst must be addressed. HT-PEMFCs do not require a complicated water management system, and they can use non-Pt catalysts as electrodes. Therefore, the international trend is gradually developing towards HT-PEMFC technology, as it does not require water management. The problems faced by HT-PEMFCs include internal local temperature, flow rate, and pressure inhomogeneity, which will accelerate the aging of membrane electrode assembly (MEA) membrane materials and lead to serious degradation of fuel cell performance. Zou et al. [9] made sulfonated poly membrane to replace the traditional Nafion membrane, overcoming the problem of fuel cell performance deterioration due to dehydration at high temperatures. Kurnia et al. [10] simulated the inlet air flow of cathode, anode, and coolant, and summed up the relationship between total power and net power and various conditions. Laribi et al. [11] found that, when the operating temperature of the fuel cell rises from 60 °C to 140 °C, the resistance of the film increases with time, and the water content of the film decreases, so the ionic conductivity also decreases. Yang et al. [12] used platinum as a thermistor, deposited on the surface of the membrane, and calculated the surface temperature with the aid of resistance temperature calibration data. Ko et al. [13] studied the influence of working pressure on the performance of fuel cells. The study found that, under the condition of low relative humidity, the influence of water transport is more important than reaction kinetics. The water flow can be controlled by adjusting the working pressure to obtain appropriate water distribution and achieve the best performance. Sun et al. [14] used micromachining technology to fabricate a flow sensor, studying the influence of different geometric parameters on the temperature difference. Aslam et al. [15] explored the effects of different flow rates on the cell. The performance of the fuel cell decreases with the increase in flow rate, because the rate of water removal from the cathode increases with the increase in air flow rate, resulting in a decrease in the amount of water available for humidifying the membrane electrode assembly.

At present, the research on HT-PEMFCs (in this paper HT-PEMFC means operating in the range of 120 to 200 °C) mainly focuses on the proton exchange membrane. Considering that the Nafion film used in LT-PEMFCs is only suitable for conditions below 100 °C and needs to rely on water for the transfer of protons, HT-PEMFCs must use other types that can withstand high temperatures, and which contain no membrane materials that can also transfer protons in water [16,17]. Additionally, the research on channel geometry and operating conditions is mainly based on low temperature PEMFCs. Since the principles of the low temperature PEMFCs and the HT-PEMFCs are the same, the research and operating conditions of the flow channel can be extended from the low temperature PEMFCs to the HT-PEMFCs [18,19]. Jo et al. [20], through the numerical model of HT-PEMFCs, studied the cell performance under different conditions, and proved that temperature and air-fuel ratio have important effects on the cell performance. Thomas et al. [18], through the numerical model of HT-PEMFCs, proved the superiority of the newly developed flow field in mass transfer. Wu et al. [21,22,23] used the numerical model of HT-PEMFCs to study the influence of channel rectangular ribs and achieve the best performance.

HT-PEMFCs currently use external sensors to measure various physical quantities. This research intends to use micro-electromechanical system (MEMS) technology to develop a flexible three-in-one microsensor that can withstand high-temperature electrochemical environments, observe three important physical quantities in real time, and provide real internal data. In the past, there has also been research conducted on the application of MEMS technology in fuel cells [24,25,26,27]. The information of high-temperature PEMFCs can achieve the purpose of improving its performance and prolonging its service life. Due to its extremely thin thickness, it can be well embedded in the internal flow channel of HT-PEMFCs to achieve real time microscopic monitoring.

## 2. Sensing Principle of Flexible Three-in-One Microsensor

### 2.1. Sensing Principle of Micro Temperature Sensor

The sensing principle of the micro-temperature sensor is: when the ambient temperature increases, the gold has a positive temperature coefficient (PTC) characteristic, and the resistance value increases as the temperature increases. This characteristic is due to the “resistance-temperature coefficient” (TCR) of the conductor, which is defined in Equation (1).

(1) $$\alpha =\frac{1}{\rho 0}\frac{d\rho }{dT}$$

where *α* is the resistance value-temperature coefficient; *ρ* is thermal resistivity; *ρ*_0_ is the thermal resistivity at 0 °C.

### 2.2. Sensing Principle of Micro-Flow Sensor

The principle of the hot wire flow sensor is to measure the flow rate in a way that the heat consumption rate of the heater is positively correlated with the fluid velocity. The structure of the hot wire flow sensor is a heater, which forms a stable temperature field. In the next step, it is placed in the flowing flow field, and the temperature field generated by the heater will change with the forced thermal convection of the fluid. At this time, the resistance value of the heater will increase due to the heat taken away, so that the resistance value will also decrease.

### 2.3. Sensing Principle of Micro Pressure Sensor

The capacitive pressure sensor has a non-conductive dielectric layer sandwiched between two parallel electrodes, and the formula for calculating the capacitance value is shown in Equation (2).

(2) $$\Delta C=\varepsilon_{r}\;\varepsilon_{0}\;\frac{A}{\Delta d}$$

where *ε*_r_ is the dielectric constant of the material, *ε*_0_ is a constant of 8.854 × 10^−12^ (F/m), *A* is the projected overlap area of the two parallel electrodes, and Δ*d* is the change in the vertical distance between the two parallel electrodes. From Equation (2), it can be known that the dielectric constant and the projected area of two parallel electrodes only affect the initial capacitance value, and the main change of capacitance is the change of distance between two parallel electrodes.

## 3. Manufacturing Process of Flexible Three-in-One Microsensor

**Figure 1:**
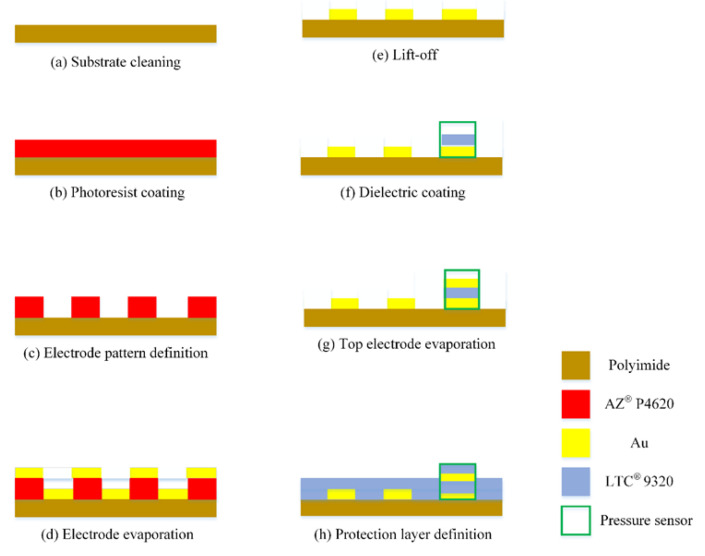
Manufacturing process of flexible three-in-one microsensor.

**Figure 2:**
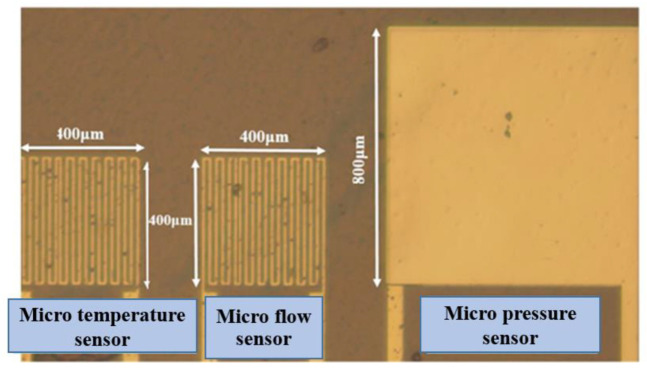
Optical micrograph of flexible three-in-one microsensor size and layout.

The flexible substrate selected in this study has many advantages, such as high temperature resistance, compression resistance, high flexibility, and good durability, as is the case with polyimide (PI) [28]. The protective layer uses Fujifilm Electronic Materials USA, Inc. LTC^®^ 9320 (Phoenix, AZ, USA) liquid polyimide; the overall process is based on surface micromachining, including deposition, Lithography, and lift-off. In the past, laboratories used the wet etching process, but there were always the problems resulting from over-etching, which caused the high resistance and unstable quality of each batch. Therefore, this research switched to the lift-off process to reduce the over-etching, making the quality of each batch of micro-sensors more stable. The manufacturing process and optical microscope photos of the flexible three-in-one microsensor, developed by using MEMS technology, are shown in Figure 1 and Figure 2. The flexible three-in-one microsensor is small in size and high in sensitivity, and, as a result, it can be embedded in the internal real-time microscopic diagnosis and analysis without affecting the operation of HT-PEMFCs.

## 4. Calibration of Flexible Three-in-One Micro Sensor

In this study, the NI PXI 2575 digital capture device from National Instruments (NI) is used to capture the resistance, current, and capacitance values of micro temperature, strain, flow, and pressure sensors in real time, and we use LabVIEW system design software to control the acquisition action of the system. After the acquired data is calculated and analyzed, a calibration curve can be drawn to confirm its reliability.

### 4.1. Calibration of Micro Temperature Sensor

**Figure 3:**
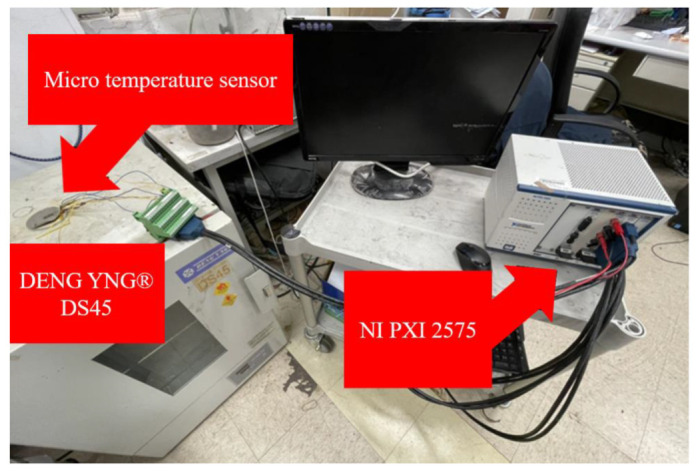
Temperature correction test.

**Figure 4:**
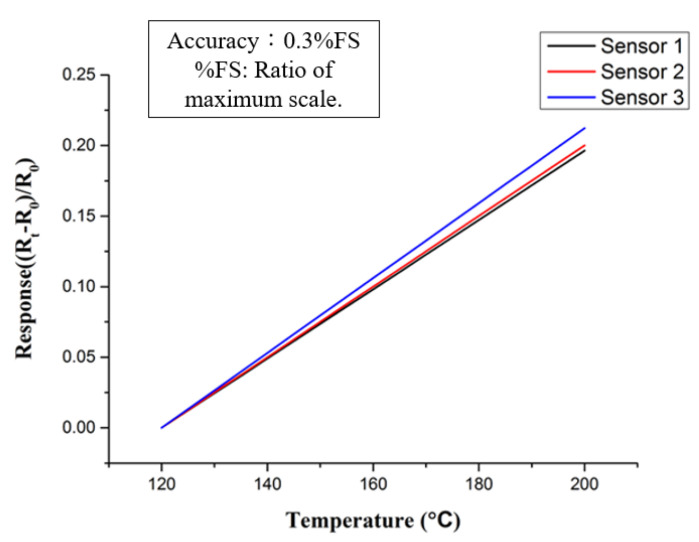
Dimensionless calibration curve of micro temperature sensor.

Three-in-one micro sensors need to be embedded in high-temperature fuel cells in practical applications. Therefore, the micro-sensor is embedded in the high-temperature fuel cells during temperature correction, and the actual application environment is simulated with a torque combination of 25 kg·cm. Resulting from the high temperature correction range being 100 °C, and because the humidity is zero, there is no need to consider the influence of humidity. Therefore, we use the DENG YNG^®^ DS45 constant temperature oven owned by our laboratory to gradually increase the temperature at 5 °C intervals to complete the temperature correction. In order to reduce the temperature error, the micro sensor is placed close to the oven thermocouple, and the overall calibration test setup is shown in Figure 3. We connect the miniature sensor to NI PXI 2575 and heat it, staying at each temperature point for 3 min, capturing the value within 3 min, and taking the average. This action loops three measured values to calculate the average value, and the result is made into a dimensionless graph. This dimensionless graph is the calibration curve of the miniature temperature sensor as shown in Figure 4. With the acquired data, each can be calculated at the same time. According to the TCR and sensitivity of the microsensor, the calculated TCR is about 2777 ppm/°C, and the sensitivity is about 1.8517 Ω/°C.

### 4.2. Calibration of Micro Flow Sensor

**Figure 5:**
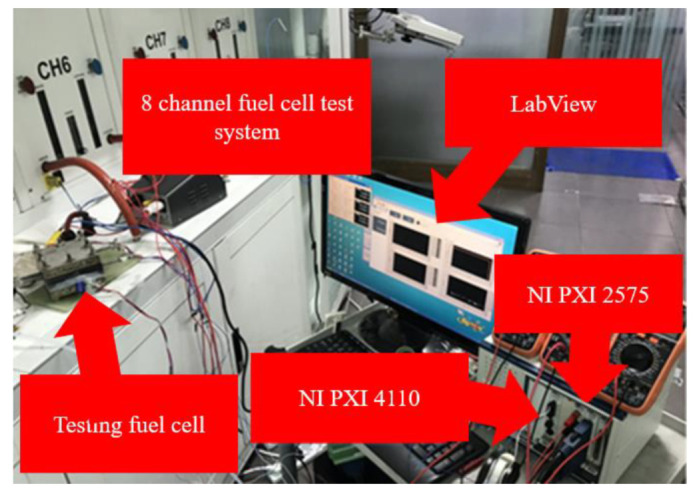
Flow correction test.

**Figure 6:**
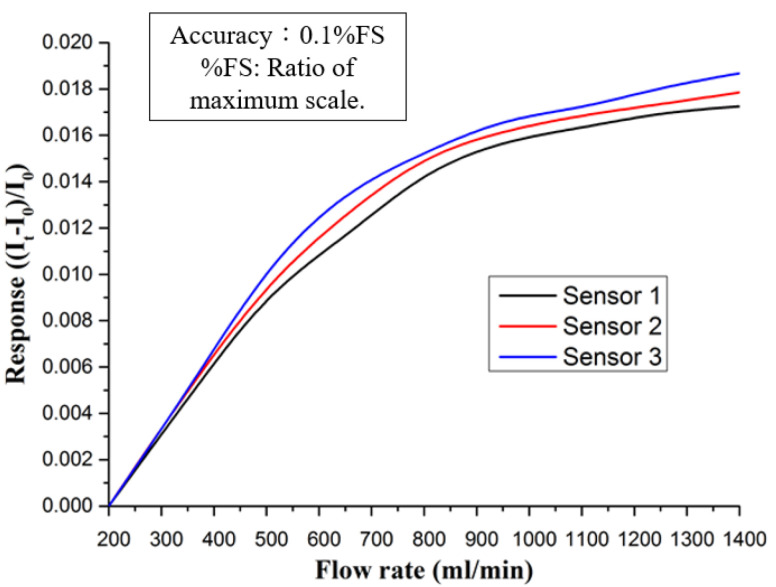
Dimensionless calibration curve of micro flow sensor.

The flow correction test setup is shown in Figure 5. First, the micro flow sensor is aligned with the flow channel, and the fuel cell is combined with a torque of 25 kg·cm. In the next step, the NI PXI 4110 power supply, the NI PXI 2575 data capture device, and the micro flow sensor are combined. It uses NI PXI 4110 power supply to output 3 V constant voltage to the micro flow sensor. The flow calibration range is from 200 to 1400 mL/min, and the measurement is performed at an interval of 150 mL/min. The micro flow sensor is dimensionless calibration. The curve is shown in Figure 6.

### 4.3. Calibration of Micro Pressure Sensor

**Figure 7:**
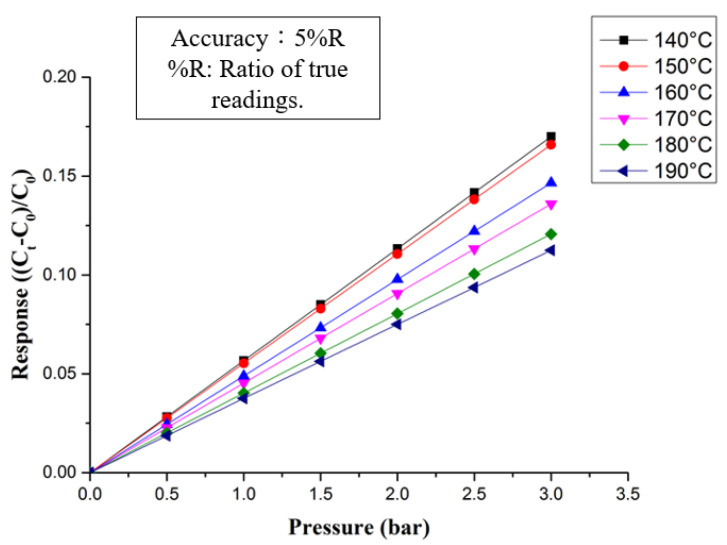
Dimensionless calibration curve of micro pressure sensor.

The micro pressure sensor uses a Druck-DPI 530 pressure controller to apply a fixed pressure to the micro pressure sensor; it has a pressure measurement range of 0 to 300 psig with an accuracy of ±0.1% FS. At the same time, a Wayne Kerr Electronics 4230 LCR meter is used to collect capacitance data. The instrument can measure the range of capacitance values. It is 0.01pF~1F, and its accuracy is ±0.1%; in the high temperature and stable environment of the micro pressure sensor depth sounder HT-PEMFC, the dielectric constant of the material will change due to the temperature of different methods of dielectric improvement, which is almost Real operation, and, as a result, using different micro pressure measuring devices at 140 °C to 190 °C, the smaller the junction is, and the more temperature and temperature changes can be sensed, because the dielectric layer is made of PI polymer material, it is necessary to repeat the final pressure. After unloading, the capacitance value will tend to be stable, and the actual number of times has not been determined. The final result of the dimensionless micro-sensor pressure sensor is shown in Figure 7.

## 5. Assembly Design of HT-PEMFC

**Figure 8:**
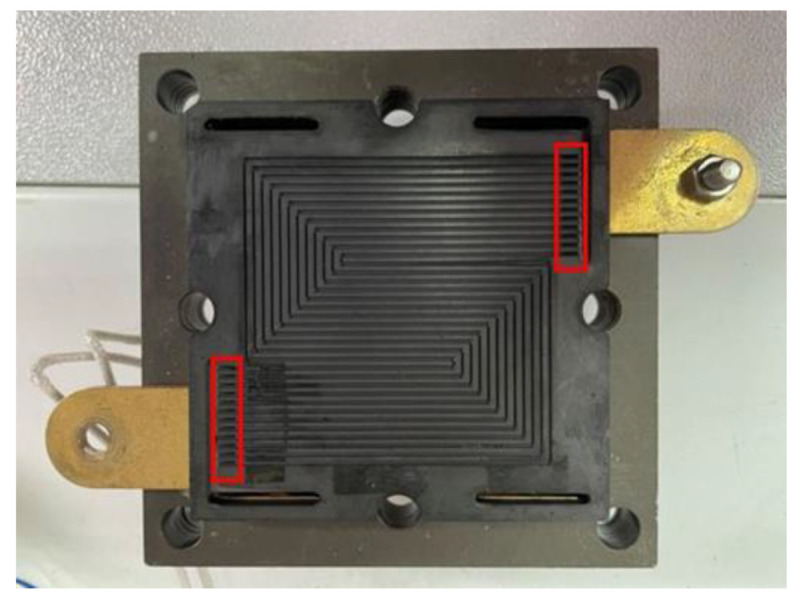
Graphite serpentine runner plate.

**Figure 9:**
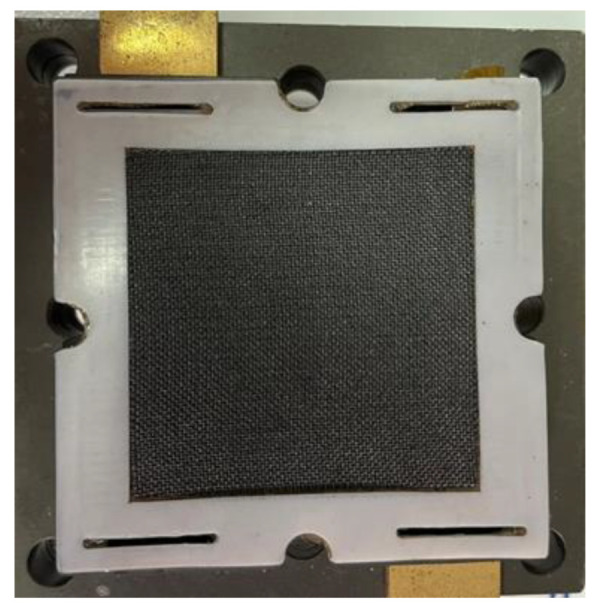
Commercially purchased high temperature MEA.

The HT-PEMFC system used in this study was provided by the Fuel Cell Center of Yuan Ze University. This HT-PEMFC system uses a graphite serpentine runner plate (Figure 8); MEA is made of commercially available Advent TPS^®^ MEA (Figure 9). High temperature proton exchange membrane (HT-PEM) fuel cells operate most effectively at temperatures ranging from 160 °C to 200 °C [29]. Advent TPS^®^ MEAs operate from 120 °C to 200 °C with good acid management and rugged membrane under differential pressure. The detailed specifications of MEA are shown in Table 1. The reaction area of the HT-PEMFC is 31.4 cm^2^ and operating environment is set 120 °C to 200 °C. In order to stabilize the HT-PEMFC performance and to avoid the fuel gas leaking during the testing process—the graphite bipolar plate breaks if the closing pressure is too high, and the gas leaks if the closing pressure is too low—this study uses closing pressure of 25 kg/cm^2^ to close the cell uniformly.

## 6. Test and Calibration of Flexible Three-in-One Microsensor

**Figure 10:**
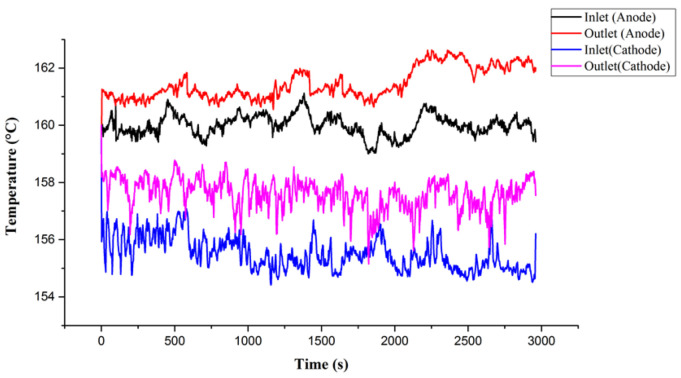
Temperature changes of HT-PEMFCs at different positions.

**Figure 11:**
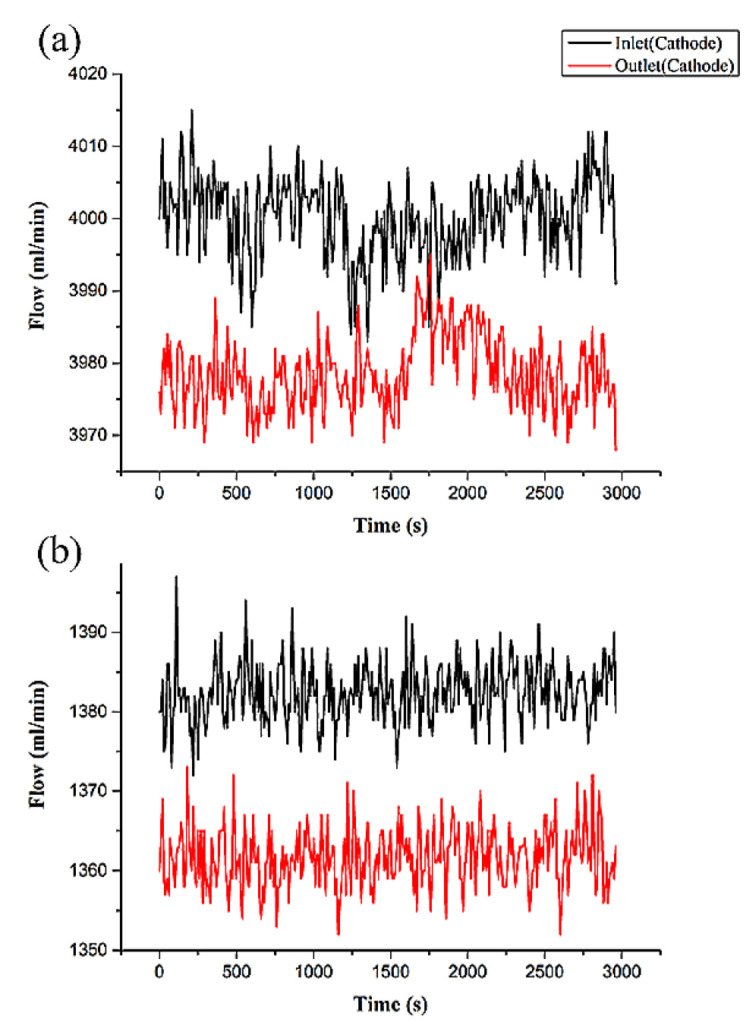
Flow changes of inlet and outlet on (**a**) anode and (**b**) cathode of HT-PEMFCs.

**Figure 12:**
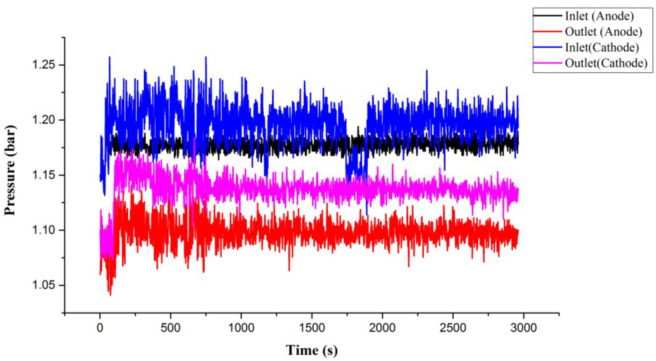
Pressure variation of HT-PEMFCs at different positions.

By using the high temperature fuel cell test machine and NI data extractor, the internal information extraction and microscopic diagnosis analysis of the HT-PEMFC were carried out, and the local temperature flow rate, pressure changes, and distributions in the HT-PEMFC were monitored and discussed under constant current conditions. The reaction area of the HT-PEMFC is 31.4 cm^2^. At the operating temperature of 160 °C, different un-humidified gas flows of anode flow rate (H_2_) and cathode flow rate (air) are given, and a constant current (0.8 A/cm^2^) is given. Through the NI PXI 2575 data extractor, the local physical quantities inside the high-temperature fuel cell were discovered, discussed, and analyzed. Detailed operating conditions are shown in Table 2. The observed temperature, flow, and force changes are shown in Figure 10, Figure 11 and Figure 12. First, we observe the temperature change (Figure 10). It is noted that the inlet temperature is lower than the outlet temperature, which is because the gas is heated by the flow channel when it passes through the flow channel, and, resultingly, the heat dissipation capacity is poor at the outlet position, and the outlet temperature is higher than the inlet temperature. In the next step, we discuss the change of flow (Figure 11). The flow data is used to judge whether the flow channel design is good or not according to the difference between the inlet and outlet. According to Faraday’s law, the overall airflow balance (anode + cathode) of the battery is abnormal (about −40 mlpm). Under normal conditions, the anodes are balanced. Hydrogen consumption = 25.12 Amp × 6.96 mlpm/Amp = 175 mL/min. The anode flow rate should be 175 mlpm lower than the inlet flow rate. In the next step, we discuss cathode balance. Oxygen is consumed and water vapor is formed at the cathode. Oxygen consumption = 25.12 Amp × 3.48 mlpm/Amp = 87.5 mlpm, the gas flow of the whole cell is balanced (anode + cathode), −175 mlpm (anode) + 87.5 (cathode) = −87.5 mlpm, which does not match the sensor data. It is speculated that the air-tight and leak-proof design of the cell causes the flow difference to be smaller than normal. Finally, we discuss the internal pressure distribution of HT-PEMFCs (Figure 12). Pressure is also an important physical quantity for HT-PEMFCs. When external air is continuously supplied into the cell, the cell pressure should be greater than one atmosphere, but, if there is a gas leak inside the cell, the internal pressure will remain at one atmosphere without change. According to the results of this experiment, it is observed that the pressure at the inlet is affected by the gas flow, resulting in a higher and unstable pressure.

## 7. Conclusions

This study successfully developed a flexible three-in-one microsensor, which is small in size and high in sensitivity. It can be embedded in the internal microscopic diagnosis and analysis of local temperature, flow rate, and pressure distribution in real time without affecting the operation of HT-PEMFCs. Preliminary observation and measurement data show that the HT-PEMFC has poor heat dissipation capacity at the anode and the outlet, the flow difference between the outlet and the inlet are not matched with normal conditions, and, resultingly, it may be necessary to redesign the flow path in order to improve flow problems, and to ensure that the pressure is maintained at about one atmosphere. It is observed that HT-PEMFCs do not have external gas leakage. Our main work is to sense the internal physical quantity data of the HT-PEMFC runtime. Finally, the aforementioned, successfully measured, data is provided to the developer of the HT-PEMFC, in order to assist the developer in creating an improved design in response to the problems, leading to the improved performance of HT-PEMFCs.

## Figures and Tables

**Table 1:** Commercial Advent TPS^®^ MEA detailed specifications.

Thickness	60~65 μm
Conductivity	8 × 10^−2^ S/cm
Reaction area	31.4 cm^2^
Operating temperature	120~200 °C
GDL thickness	300 μm

**Table 2:** Test conditions of HT-PEMFCs.

Items	Conditions
Battery temperature (°C)	160
Anode terminal flow (H_2_)(lspm)	2
Cathode terminal flow (Air)(lspm)	4
Gas temperature	Room temperature
Constant current (A/cm^2^)	0.8
Reaction area (cm^2^)	31.4

## References

[1] Ma W., Zhao C., Yang J., Ni J., Wang S., Zhang N., Lin H., Wang J., Zhang G., Li Q. (2012). Cross-linked aromatic cationic polymer electrolytes with enhanced stability for high temperature fuel cell applications. Energy Environ. Sci..

[2] Wang P., Liu Z., Li X., Peng J., Hu W., Liu B. (2019). Toward enhanced conductivity of high-temperature proton exchange membranes: Development of novel PIM-1 reinforced PBI alloy membranes. Chem. Commun..

[3] Li N., Leng Y., Hickner M.A., Wang C.Y. (2013). Highly stable, anion conductive, comb-shaped copolymers for alkaline fuel cells. J. Am. Chem. Soc..

[4] Zhang N., Zhao C., Ma W., Wang S., Wang B., Zhang G., Li X., Na H. (2014). Macromolecular covalently cross-linked quaternary ammonium poly with polybenzimidazole for anhydrous high temperature proton exchange membranes. Polym. Chem..

[5] Bai H., Peng H., Xiang Y., Zhang J., Wang H., Lu S., Zhuang L. (2019). Poly(arylene piperidine)s with phosphoric acid doping as high temperature polymer electrolyte membrane for durable, high-performance fuel cells. J. Power Sources.

[6] Carrette L., Friedrich K.A., Stimming U. (2001). Fuel cells—Fundamentals and applications. Fuel Cells.

[7] Krastev V.K., Falcucci G., Jannelli E., Minutillo M., Cozzolino R. (2014). 3D CFD modeling and experimental characterization of HT PEM fuel cells at different anode gas compositions. Int. J. Hydrogen Energy.

[8] Chandan A., Hattenberger M., El-Kharouf A., Du S., Dhir A., Self V., Pollet B.G., Ingram A., Bujalski W. (2013). High temperature (HT) polymer electrolyte membrane fuel cells (PEMFC)—A review. J. Power Sources.

[9] Zou Y., Yang M., Liu G., Xu C. (2020). Sulfonated poly (fluorenyl ether ketone nitrile) membranes used for high temperature PEM fuel cell. Heliyon.

[10] Kurnia J.C., Sasmito A.P., Shamim T. (2017). Performance evaluation of a PEM fuel cell stack with variable inlet flows under simulated driving cycle conditions. Appl. Energy.

[11] Laribi S., Mammar K., Sahliac Y., Koussaa K. (2018). Air supply temperature impact on the PEMFC impedance. J. Energy Storage.

[12] Yang K., Yang Q., Zhu X., Wang H., Zhu T., Liu J. (2020). A molecular dynamics simulation on the static calibration test of a revised thin-film thermopile heat-flux sensor. Measurement.

[13] Ko D., Doh S., Park H.S., Kim M.H. (2017). Investigation of the effect of operating pressure on the performance of proton exchange membrane fuel cell: In the aspect of water distribution. Renew. Energy.

[14] Sun Z., Shen Y., Yuan C., Li X. (2019). Influence of contamination on measurement accuracy of the calorimetric air flow sensor. Measurement.

[15] Aslam R.M., Ingham D.B., Ismail M.S., Hughes K.J., Ma L., Pourkashanian M. (2019). Simultaneous thermal and visual imagine of liquid water of the PEM fuel cell channels. J. Energy Inst..

[16] Ferng Y., Su A., Hou J. (2014). Parametric investigation to enhance the performance of a PBI-based high-temperature PEMFC. Energy Convers. Manag..

[17] Su A., Ferng Y., Hou J., Yu T. (2012). Experimental and numerical investigations of the effects of PBI loading and operating temperature on a high-temperature PEMFC. Int. J. Hydrogen Energy.

[18] Taccani R., Zuliani N. (2011). Effect of flow field design on performances of high temperature PEM fuel cells: Experimental analysis. Int. J. Hydrogen Energy.

[19] Lobato J., Cañizares P., Rodrigo M.A., Pinar F.J., Mena E., Úbeda D. (2010). Three-dimensional model of a 50 cm2 high temperature PEM fuel cell. Study of the flow channel geometry influence. Int. J. Hydrogen Energy.

[20] Jo A., Oh K., Lee J., Han D., Kim D., Kim J., Kim B., Kim J., Park D., Kim M. (2017). Modeling and analysis of a 5 kWe HT-PEMFC system for residential heat and power generation. Int. J. Hydrogen Energy.

[21] Thomas S., Bates A., Park S., Sahu A., Lee S.C., Son B.R., Kim J.G., Lee D.-H. (2016). An experimental and simulation study of novel channel designs for open-cathode high-temperature polymer electrolyte membrane fuel cells. Appl. Energy.

[22] Wu H.-W., Kang D.-Y., Perng S.-W. (2017). Effect of Rectangular Ribs in the Flow Channels of HTPEM Fuel Cell by a Three-dimensional Model. Energy Procedia.

[23] Wu H.-W., Ku H.-W. (2011). The optimal parameters estimation for rectangular cylinders installed transversely in the flow channel of PEMFC from a three-dimensional PEMFC model and the Taguchi method. Appl. Energy.

[24] Otsuki Y., Shigemasa K., Araki T. (2020). Measurement of temperature difference on catalyst layer surface under rib and channel in PEFC using micro sensors. Int. J. Heat Mass Transf..

[25] Sparks D., Kawaguchi K., Yasuda M., Riley D., Cruz V., Tran N., Chimbayo A., Najafi N. (2008). Embedded MEMS-based concentration sensor for fuel cell and biofuel applications. Sens. Actuators A Phys..

[26] Yamazaki Y. (2004). Application of MEMS technology to micro fuel cells. Electrochim. Acta.

[27] Wang H., Morando S., Gaillard A., Hissel D. (2021). Sensor development and optimization for a proton exchange membrane fuel cell system in automotive applications. J. Power Sources.

[28] Baloda S., Alam Ansari Z., Singh S., Gupta N. (2020). Development and Analysis of Graphene Nanoplatelets (GNPs)-Based Flexible Strain Sensor for Health Monitoring Applications. IEEE Sens. J..

[29] Waller M.G., Walluk M.R., Trabold T.A. (2016). Performance of high temperature PEM fuel cell materials. Part 1: Effects of temperature, pressure and anode dilution. Int. J. Hydrogen Energy.

